# Exploring the Agricultural Applications of Microbial Melanin

**DOI:** 10.3390/microorganisms12071352

**Published:** 2024-07-02

**Authors:** Patricio Muñoz-Torres, Steffany Cárdenas-Ninasivincha, Yola Aguilar

**Affiliations:** Laboratorio de Patología Vegetal y Bioproductos, Facultad de Ciencias Agronómicas, Universidad de Tarapacá, Av. General Velásquez 1775, Arica 1000000, Chile; sfcninasivincha@gmail.com (S.C.-N.); catacorayol35@gmail.com (Y.A.)

**Keywords:** melanin, microbial pigments, agriculture, bioinsecticide, antimicrobial, plant growth promotion, bioremediation

## Abstract

Microbial melanins are a group of pigments with protective effects against harsh conditions, showing fascinating photoprotective activities, mainly due to their capability to absorb UV radiation. In bacteria, they are produced by the oxidation of L-tyrosine, generating eumelanin and pheomelanin. Meanwhile, allomelanin is produced by fungi through the decarboxylative condensation of malonyl-CoA. Moreover, melanins possess antioxidant and antimicrobial activities, revealing significant properties that can be used in different industries, such as cosmetic, pharmaceutical, and agronomical. In agriculture, melanins have potential applications, including the development of new biological products based on this pigment for the biocontrol of phytopathogenic fungi and bacteria to reduce the excessive and toxic levels of agrochemicals used in fields. Furthermore, there are possibilities to develop and improve new bio-based pesticides that control pest insects through the use of melanin-producing and toxin-producing *Bacillus thuringiensis* or through the application of melanin to insecticidal proteins to generate a new product with improved resistance to UV radiation that can then be applied to the plants. Melanins and melanin-producing bacteria have potential applications in agriculture due to their ability to improve plant growth. Finally, the bioremediation of water and soils is possible through the application of melanins to polluted soils and water, removing synthetic dyes and toxic metals.

## 1. Introduction

Agriculture is facing the challenge of providing nutritious and innocuous food to a population that is growing from 7.5 billion people to ~10 billion by 2050 [1]. Furthermore, inadequate agricultural methods, adverse natural conditions, substandard soil management, and the excessive use of agrochemicals have diminished arable land availability, leading to an escalation in salinization processes and the pollution of water and ecosystems [2]. In the face of this scenario, it is crucial to embrace practices that sustain soil productivity, viability, and quality while safeguarding the environment and its resources, ensuring their availability for future generations to cultivate plant-based foods [3].

Under this scheme, researchers are increasingly turning their attention to the development of new bio-based products for sustainable solutions to pressing challenges. Among these biotechnological solutions, microbial melanin emerges as a dark, enigmatic pigment with profound implications for agriculture. Far beyond its role in coloring fungi and bacteria, microbial melanin harbors several properties that promise to revolutionize agricultural practices [4].

Melanin is a general term employed to name a group of heterogeneous pigments produced by different organisms and microorganisms. This heterogenicity is due to the ubiquitous sources of melanin, leading to differences in composition, size, color, and function. However, similar characteristics are common in different melanins, including a high negative charge, high molecular weight, and hydrophobic nature. This pigment is insoluble in most solvents and is resistant to chemical degradation. Strong bases are commonly used to dissolve melanin; however, the chemical structure of melanin can be altered, and it is possible to break the polymer into fragments [5,6].

**Figure 1:**
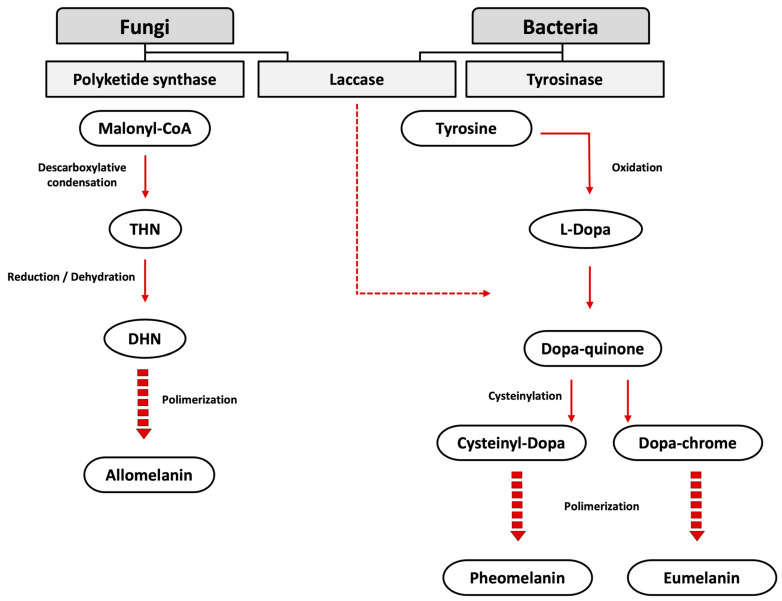
Biosynthesis of microbial melanins. Modified from Singh et al. [14].

According to Solano [6], melanin is a “heterogeneous polymer derived from the oxidation of phenolic or indolic compounds and subsequent polymerization of intermediate phenols and their resulting quinones”. Based on their chemical structure, melanins can be classified into eumelanin (black to dark brown melanin produced through the oxidative polymerization of derivatives of tyrosine), pheomelanin (differs from eumelanin due to the presence of sulfhydryl groups in its chemical structure), neuromelanin (produced in human neurons via the oxidation of catecholamine precursors and dopamine), and allomelanin (non-nitrogenous melanins produced from different chatecolic and dihydroxynaphtalene precursors) [4,6,7,8]. In microorganisms, molecular precursor units of eumelanin consist of indole-type units derived from L-tyrosine or L-Dopa oxidation. Meanwhile, pheomelanin is produced via the oxidative polymerization of cysteinyl conjugates of L-Dopa via benzothiazine intermediates. For allomelanin, its production occurs via the oxidation of nitrogen-free diphenols, such as catechol, 1,8-dihydroxynaphtalene, and γ-glutaminyl-3,4-dihydroxybenzene. The production of microbial melanins is catalyzed by three enzymes as follows: tyrosinases, present in bacteria; polyketide synthase, in fungi; and laccases, present in bacteria and fungi (Figure 1) [4,9,10].

In microorganisms, melanin possesses protective effects against environmental stress conditions. Melanin shows photoprotective properties due to its capacity to absorb UV radiation, dissipating up to 90% of the absorbed energy from sunlight radiation, such as heat [6]. Moreover, melanin is able to absorb other types of radiation, including gamma radiation, as described by Dadachova and Casadevall in 2008 [11]. Authors suggested that highly melanized fungi showed enhanced growth during their exposure to Chernobyl ionizing radiation. Furthermore, melanin confers resistance to a variety of stress conditions, including high temperatures or desiccation [6]. In addition, melanin performs the quenching of free radicals [12], participates in enhancing the virulence mechanisms in pathogenic bacteria and fungi [13], and possesses antibacterial properties [14]. Other potential applications have been described for melanins such as semiconductors, metal chelators, optical imagers, cosmetics, and pharmaceuticals, among others [14,15,16].

Studies regarding the application of microbial melanins in agriculture are scarce. This review summarizes the biosynthesis of melanin produced by bacteria and fungi, emphasizing the key enzymes involved in this pigment’s synthesis pathway. Moreover, this review describes the application of melanin and melanin-producing microorganisms in agriculture, including the biological control of phytopathogenic fungi and bacteria, the production of biopesticides, and the use of this pigment in plant growth promotion. Furthermore, the disadvantages and limitations of using melanin in agriculture are discussed.

## 2. Microbial Biosynthesis of Melanin

The microbial biosynthesis of melanin occurs via the oxidative polymerization of polyphenolic compounds through two pathways, which produce different types of pigment. This process is catalyzed by different enzymes, including tyrosinases, laccases, and polyketide synthase (Figure 1) [14,16,17,18].

The general pathway uses two precursor molecules as follows: tyrosine and malonyl CoA. When malonyl CoA reacts through polyketide synthases or some laccases, it gives rise to DHN-melanin. Meanwhile, tyrosinase and laccase produce DOPA-melanin when tyrosine acts as a substrate [14,17].

The fungal biosynthesis of melanin involves the activity of the polyketide synthase enzyme, which uses acetyl CoA or malonyl CoA as a substrate and forms 1,3,6,8-tetrahydroxynaphthalene (THN). As a result of the steps of reduction and dehydration reactions, the intermediate 1,8-dihydroxynaphthalene (DHN) is formed. The polymerization of DHN leads to the formation of melanin [7].

In the bacterial DHN-melanin pathway, THN is formed through condensing five molecules of malonyl CoA in a sequential step catalyzed by the enzyme polyketide synthase type III. Two molecules of THN dimerize, generating hexahydroxyperylenequinone (HPQ) in a step catalyzed by a cytochrome P450 enzyme, which is co-transcribed with the polyketide synthase [14].

For the pathway of l-3,4-dihyroxyphenylalanine (L-Dopa), there are two possible substrates, L-Dopa or tyrosine. When L-Dopa is the precursor, the enzyme laccase catalyzes its oxidation to dopaquinone. If tyrosine is the substrate, it is transformed to L-Dopa and then to dopaquinone. The enzyme tyrosinase catalyzes both steps. Dopaquinone is highly reactive, and cyclodopa is formed by an intramolecular nucleophilic addition by the amino group. Cyclodopa is then oxidized to form dopachrome. The tautomerization of dopachrome generates dihydroxyindoles that then polymerize into melanin [7].

### 2.1. Enzymes Involved in the Microbial Biosynthesis of Melanin

#### 2.1.1. Polyketide Synthase

Polyketide synthases (PKSs) are enzymes widely distributed in bacteria, fungi, and plants. They are involved in different processes that produce secondary metabolites, including pigments, antibiotics, and toxins [14]. Type III PKSs catalyze the iterative condensation reactions on a starter molecule utilizing an acetyl unit derived from the decarboxylative condensation of malonyl-CoA. Once expanded to a preferred length, dictated by the shape, volume, and chemical properties of the type III PKS active-site cavity, the tautomeric keto-enol intermediate undergoes one or more of three cyclization reactions, namely Claisen condensation, aldol condensation, or lactonization [19,20].

In fungi, the biosynthesis of DHN-melanin is started by a PKS, which catalyzes the formation of THN from acetyl CoA, followed by catalyzed reactions of reduction and dehydration to form DHN, which is finally converted to melanin in a reaction catalyzed by laccases [21].

In *Penicillium marneffei*, the melanin-biosynthesis gene cluster spans a region of 25.3 kb in the fungus’s genome. This cluster includes the *alb1* gene codifying the PKS enzyme. This cluster could contribute to the fungus’s virulence through decreasing its susceptibility to killing by hydrogen peroxide. It was tested by knocking down the *alb1* gene, resulting in phenotypic changes, including a loss of pigmentation, a decrease in virulence, and a decrease in the resistance to killing by hydrogen peroxide [22].

#### 2.1.2. Tyrosinase

Tyrosinases are copper-dependent enzymes involved in the hydroxylation of tyrosine to form L-Dopa and its subsequent oxidation to *o*-dopaquinone [23]. This compound suffers spontaneous cyclization to indole quinone, which polymerizes, forming DOPA-melanins (eumelanin) [18,24].

It has been described that many bacterial genomes codify for more than one operon containing tyrosinase genes [21]. For example, there are two operons in streptomycetes, named *melC* and *melD,* which are responsible for producing two tyrosinases, MelC2 and MelD2, respectively. The *melC* operon is only present in the melanin-producing *Streptomyces* strains. The production of melanin is associated with MelC2, which has been shown to participate in the uptake of catechols via the oxidizing of them to their respective quinones [25]. The tyrosinase MelC2 remains inactive until it is secreted and activated by MelC1 (chaperone for secretion and copper incorporation into the active site of MelC2). After the transportation, MelC1 dissociates from MelC2, which activates tyrosinase activity [26].

In general, bacterial tyrosinases have an optimal pH of ~7.5 and an optimal temperature of ~40 °C. These enzymes are monomeric, with a molecular mass ranging from 20 to 60 kDa [21]. However, tyrosinases with unique characteristics have been described. For example, the dimeric tyrosinase from *Thermomicrobium roseum* is composed of two identical subunits, each with a molecular mass of 43 kDa. Moreover, the enzyme’s activity is optimal at 70 °C and pH 9.5 [27].

The presence of tyrosinases involved in the biosynthesis of melanin in Gram-positive and Gram-negative bacteria has been described, including *Sinorhizobium meliloti* [28], *Bacillus thuringiensis* [29], *Bacillus megaterium* [30], *Bacillus licheniformis* [31], and *Vibrio nigripulchritudo* [32].

#### 2.1.3. Laccase

Laccases are blue multicopper oxidases involved in the oxidation of multiple substrates, including polyphenols, methoxy-substituted phenols, and diamines [12,33]. They have been described to participate in the biosynthesis of DHN-melanin and Dopa-melanin. Laccases participate in the one-step oxidation of dihydroxy phenols to quinones, unlike tyrosinases, which catalyze the reaction in two steps [34].

Bacterial laccases, involved in melanin synthesis, are present in *S. meliloti* [35], *Bacillus subtilis* [36], and *Bacillus weihenstephanensis* [37]. Furthermore, some bacteria have been shown to exhibit tyrosinase and laccase activity, as in *Pseudomonas putida* F6 [38], *Alteromonas* sp. [39], and *Bacillus* sp. [40]. The presence of laccase activity in fungi has been described in *Scleromitrula shiraiana* [41], *Cryptococcus neoformans* [42], *Lentinula edodes* [43], and *Talaromyces marneffei* [44].

## 3. Agricultural Applications of Melanin

### 3.1. Biological Control of Phytopathogenic Fungi and Bacteria

The application of microbial melanin has generated significant interest in several fields, such as medicine, the environment [45], and, more recently, agriculture. In this last area, the application of microbial melanin has exceptional potential since it contributes significantly to increasing iron bioavailability via reducing the ferric ions to their ferrous form [26]. This process is pivotal for siderophores, a metabolite that stimulates the defensive response of plants against different phytopathogenic microorganisms [46]. Therefore, applying microbial melanin in agriculture could be a promising strategy to strengthen the health and resistance of crops against diseases.

Regarding melanin-producing bacteria, the *Pseudomonas* genus and the bacterium *Legionella pneumophila* can produce pyomelanin, which is associated with the ferric reductase activity [46]. The strain U7 of the bacterium *Pseudomonas balearica* showed antibacterial activity against the phytopathogenic bacteria *Erwinia chrysanthemi*, which causes corn stalk rot, and *Erwinia carotovora*, the etiological agent of potato tuber rot [47]. Likewise, the bacterium *Pseudomonas putida* strain black showed the ability to inhibit the growth of phytopathogenic bacteria, such as *Erwinia amylovora*, *Xanthomonas vesicatoria*, *Clavibacter michiganensis* subsp. *michiganenesis*, *Pseudomonas syringae* pv. *tomato,* and *Pseudomonas syringae* pv. *syringae*, which affect tomato and bean crops. *In planta* and field assays are necessary to assess whether the foliar application of *P. putida* strain black can effectively reduce the impact of diseases caused by phytopathogens antagonized in vitro. Additionally, given that this strain was isolated from a rhizosphere, it is plausible that this isolate could colonize a host plant and promote its growth through mechanisms such as phosphate solubilization or increased iron availability. Moreover, it might enhance the induced systemic resistance (ISR) and improve plant protection [48].

Another relevant bacterium in this context is *Azotobacter chroococcum*, which synthesizes melanin through the oxidation of tyrosine via the enzyme tyrosinase, which requires the presence of copper. The melanin produced by *A. chroococcum* showed effectiveness in inhibiting the growth of phytopathogenic fungi, such as *Aspergillus*, *Alternaria*, *Fusarium oxysporum*, and *Rhizoctonia solani* [49], thus contributing to the protection of agricultural crops against fungal diseases.

The antimicrobial mechanism associated with melanin has been poorly described. However, Xu et al. suggested that the intracellular melanin of the fungus *Lachnum* YM30 possesses antibacterial activity against the Gram-negative bacterium *Vibrio parahaemolyticus* and the Gram-positive bacterium *Staphylococcus aureus* through a membrane damage mechanism. The authors’ results showed that the fungal melanin significantly damaged the integrity of the cell membrane, increased the leakage of cell contents, raised the quantity of NPN uptake, and reduced the membrane potential [50].

These melanin-producing microorganisms and the pigment melanin could have the potential to improve the health of vegetable crops via reducing the incidence of phytopathogenic microorganisms in crops. However, the evidence is limited to in vitro analysis; further in vivo and in planta experiments are needed to be performed in order to validate the antimicrobial activity of melanin as a potential bioproduct designed for agricultural purposes. Despite this, the application of melanin and melanin-producing microorganisms could be an alternative for the generation of new bioproducts which are useful for agricultural purposes in reducing the use of toxic agrochemicals.

### 3.2. Biopesticide

On the one hand, it has been observed that the protein crystals, such as Cry proteins and delta-endotoxins produced by the bacterium *Bacillus thuringiensis*, are sensitive to solar and ultraviolet (UV) radiation, which can cause an effective decrease in the bioinsecticide properties of these type of proteins [51]. On the other hand, melanin, which is present in various bacteria and fungi, helps to resist solar and UV radiation, and, in addition, to provide protection against the effect of enzymatic lysis [44]. It has been demonstrated that *B. thuringiensis* produces melanin at high temperatures (42 °C), but the bacterium loses its toxic effect. However, the addition of L-Tyrosine at high temperatures allows the bacterium to resist UV radiation and maintain bioinsecticide activity by protecting the insecticidal toxins. Toxicity assays on *Heliothis armigera* suggested that the insecticidal activity of melanin-producing *B. thuringiensis* was significantly higher after UV irradiation than when melanin was not produced. This approach demonstrates an attractive alternative through utilizing the UV-protective properties of melanin produced efficiently and safely by *B. thuringiensis*. This unique method generates a UV protectant without adding exogenous materials to *B. thuringiensis* preparations, making it both ecologically safer and more cost-effective [52].

Furthermore, it has been observed that the use of Dopa-melanin produced by the bacterium *Aeromonas media* strain WS protects commercial bioinsecticides based on *B. thuringiensis* (BTI) against solar and UV radiation, which allow maintaining the insecticidal properties of this bacterium against larvae of diptera, which affect vegetable crops [51]. Moreover, DHN-melanin extracted from *Hortaea werneckii* EGYNDA08 improved the survival of *B. thuringiensis* Bt-C18 under direct UV light exposure and maximized the bacterium’s toxicity against larvae of *Spodoptera littoralis* [45].

The recombinant plasmid pIJ702 from *Streptomyces antibioticus* was cloned in *Streptomyces lividans*. This plasmid codifies for a tyrosinase gene, which allows the production of melanin in *S. lividans*. Liu et al. [53] purified the melanin and its efficacy in the protection of the mosquito (*Aedes aegypti*) larvicidal activity of *B. thuringiensis* var. *israelensis* against UV radiation. Authors observed that the δ-endotoxin of *B. thuringiensis* was sensitive to UV radiation, losing its larvicidal activity after exposure. The addition of melanin showed a photoprotective effect on the survival of *B. thuringiensis* spores and the mosquitocidal activity of the toxin following exposure to UV radiation, indicating that melanin is an excellent photoprotective agent.

These studies describe the importance of melanin in the photoprotection and effectiveness of bioinsecticides derived from *Bacillus thuringiensis* against adverse environmental conditions, where high levels of UV radiation can be harmful to the toxic activity of this bacterium. Moreover, these studies open the possibility of developing new biobased insecticides for agriculture.

### 3.3. Plant Growth Promotion

Melanin has been shown to have various effects on plant growth and development. The exogenous application of melanin has shown to have a biostimulating effect in plants, leading to increased seed germination and plant growth stimulation. Garude et al. observed that the application of melanin from the bacterium K7 to rice (*Oryza sativa*) seeds improved seed germination (82%) in comparison to the control (55%). Moreover, the treated plantlets’ shoot and root lengths were 15.3 cm and 13.1 cm, respectively. Meanwhile, the same parameters in non-treated plantlets were 11.2 cm and 8.6 cm, respectively, indicating that bacterial melanin improves the plant growth parameters of rice plants [54]. Moreover, Castro-Sowinski et al. observed that the co-inoculation of melanin-producing *Sinorhizobium meliloti* strain CE52G and *Azospirillum brasilense* strain Cd in rice plants increase the shoot and root yield above that of *A. brasilense* alone, and the co-inoculation of *A. brasilense* Cd and *S. meliloti* U143 (melanin-producing strain) increased the root dry matter of rice plants. Moreover, a deleterious effect on rice growth was observed when plants were inoculated with the non-melanin-producing strains of *S. meliloti* CE47 and CE15 [35], suggesting that melanin-producing strains could improve the plant growth promotion of rice plants.

### 3.4. Bioremediation of Soils

Pollution produced by dumping toxic compounds into soil and water is one of the leading environmental threats with negative consequences for biological diversity. These toxic compounds include heavy metals and synthetic dyes, which can be transferred from soil to plants and invertebrates and can reach high toxicological concentrations in tissues [55,56]. In this context, the application of fungal and bacterial melanin in bioremediation has exceptional potential to immobilize heavy metals, xenobiotics, and organochlorines derived from agricultural and industrial activities that affect water and soil [44,47,57]. Xenobiotics, such as synthetic dyes, can accumulate and harm the soil microbiome. This accumulation also affects plant performance through interfering with photosynthesis and growth. These dyes, present in textile effluents, are frequently discarded into water due to inefficiency in the processing and treatment of industrial waste, which causes severe pollution in both soil and water. These textile effluents can contain a wide range of highly toxic heavy metals, such as cadmium, chromium, cobalt, copper, mercury, nickel, and other elements [55,58].

Melanin-producing fungal microorganisms, such as *Phanerochaete chrysosporium*, *Bjerkandera adusta*, *Trametes versicolor*, *Phlebia radiata*, and *Pleurotus* spp., have demonstrated a remarkable ability to degrade synthetic dyes, thanks to the production of enzymes such as laccase. For example, *B. adusta* and *T. versicolor* can remove up to 95% of the dye HRB 8, while *P. chrysosporium* can mineralize various toxic dyes and other hazardous aromatic ring compounds [59]. According to Rassabina et al. [55], the melanin produced by the bacterium *Bacillus subtilis* can mitigate the toxicity of synthetic dyes, while the melanin present in the lichens *Lobaria pulmonaria* and *Lobaria retigera* proves to be effective in terms of the adsorption and retention capacity of the synthetic dyes.

The excessive and prolonged use of organochlorine pesticides for decades has generated a significant problem of soil and water contamination, which is why the bioremediation of organochlorine compounds is necessary [49]. In this sense, melanin-producing bacteria, such as *Arthrobacter* sp., *Burkholderia* sp., *Pseudomonas* sp., *Azotobacter* sp., and others present potential. These bacteria can degrade endosulfan and improve soil fertility [58]. For example, *Azotobacter chroococcum* and *Azotobacter vinelandii* have demonstrated their effectiveness in the biodegradation of endosulfan, chlorpyrifos, pendimethalin, phorate, glyphosate, carbendazim, hexachlorocyclohexane (HCH), monocrotophos, aldrin, and others [47]. Furthermore, *Pseudomonas oryzihabitans* has the ability to degrade *m*-toluene efficiently due to the catechol enzyme [60].

On the one hand, pyomelanin, produced by native soil bacterial species, can immobilize uranium in soils for prolonged periods by producing illite or goethite. On the other hand, the bacterium *Azotobacter chroococcum* shows the chelating activity of heavy metals such as cadmium, chromium, and nickel, which contributes to the remediation of contaminated soils [14,61]. As for the melanin of *Pseudomonas stutzeri*, it turned out to be a good adsorbent to impregnate iron and copper on its surface, which facilitates the removal of arsenite and arsenate from the environment [62]. In addition, this bacterium has the ability to eliminate the oxidation of heavy metals such as mercury, lead, chromium, and copper in a percentage greater than 50%, which makes it a highly effective bioremediation agent [63]. Finally, fungal melanins, when incorporated with other polymers such as polycaprolactone and polyurethane, can eliminate up to 94% of lead [4]. These discoveries highlight the relevance of bacterial and fungal melanins in the bioremediation of soils contaminated by heavy metals and other toxic compounds.

## 4. Disadvantages and Limitations of Using Melanin in Agriculture

The use of melanin in agriculture could face several limitations, including high production costs, limited availability, potential environmental impacts, stability issues, and uncertain effects on crop quality. In this section, the main disadvantages and limitations of the use of melanin in agriculture are discussed.

Melanin solubility: The solubility of melanin poses a significant challenge in agricultural applications due to its inherent insolubility in water and most organic solvents. This characteristic complicates its integration into soil and plant treatments, as effective and uniform distribution is essential for the optimal performance of agricultural amendments [64]. The inability of melanin to dissolve in common solvents limits its application methods, often necessitating the development of specialized formulations or delivery systems to enhance its bioavailability and efficacy. Moreover, the insolubility of melanin may impact its interaction with soil and plant systems, potentially reducing its ability to be absorbed and utilized by plants or soil microorganisms [65]. This can hinder its effectiveness as a biocontrol agent or soil conditioner, which relies on its proper dispersion and activity within the agricultural environment.

Addressing melanin’s solubility issues requires innovative approaches, such as modifying its chemical structure or developing nano-formulations that can improve its solubility and distribution. Additionally, comprehensive studies are needed to understand the long-term impacts of melanin application on soil health and plant growth under real agricultural conditions. Overcoming these challenges is crucial for harnessing melanin’s potential benefits in sustainable agriculture, including enhanced plant protection and soil improvement.

High production costs: The production of melanin involves resource-intensive processes that contribute to its high costs, particularly on an industrial scale. Synthesizing and extracting melanin require complex procedures and often involve expensive substrates, such as specialized culture media or precursor molecules. These factors significantly elevate production expenses, rendering large-scale manufacturing economically challenging for widespread agricultural applications [7,66]. Furthermore, the purification of melanin to attain a form that is suitable for agricultural use adds to these costs. Purification processes typically involve separation techniques to isolate melanin from other cellular components or contaminants, which requires additional equipment, labor, and energy expenditure [7]. The need for high purity is crucial in agricultural settings to ensure efficacy and minimize potentially adverse effects on soil and plants. Altogether, the combination of synthesis complexities, expensive substrates, and purification requirements contributes to the overall high production costs of melanin. Addressing these cost-related challenges is pivotal for advancing melanin-based solutions in agriculture, necessitating innovative approaches in production techniques and cost-effective strategies to enhance its feasibility and affordability for broader agricultural applications.

Limited availability: Naturally occurring melanin is inherently limited in its availability within biological sources, existing in relatively low concentrations. Although microorganisms like fungi and bacteria have the capability to produce melanin, the yields from natural sources are typically insufficient for large-scale agricultural applications without substantial biotechnological interventions. This scarcity poses significant constraints on the widespread utilization of melanin in agriculture, impacting its feasibility and driving up production costs. The challenge lies in scaling up production in order to meet agricultural demands while ensuring consistent quality and efficacy. Biotechnological approaches, such as genetic engineering or fermentation processes, are often necessary to enhance melanin production yields and efficiency. These methods involve manipulating microbial strains to optimize melanin synthesis pathways or employing controlled fermentation conditions to maximize yield. Despite advancements, achieving cost-effective and sustainable production remains a critical hurdle [66]. Overcoming these limitations requires further research and development efforts focused on improving melanin production technologies and exploring alternative sources or synthetic routes to meet agricultural needs effectively and economically. Efforts to expand melanin availability and reduce production costs are essential for realizing its potential benefits as a versatile agricultural resource.

Potential environmental impact: The potential environmental impact of melanin application in agriculture remains a subject of concern due to the limited understanding of its long-term effects. While melanin is considered biologically inert, its application could inadvertently alter the soil chemistry, potentially affecting nutrient cycling and soil fertility. Moreover, melanin residues might interact with soil microbial communities, influencing their composition and function, which are critical for ecosystem stability and plant health. The impact on non-target organisms, such as beneficial insects or soil fauna, remains uncertain, raising concerns about the unintended consequences on biodiversity and ecosystem dynamics. Furthermore, the persistence of melanin in the environment and its degradation products could pose challenges in assessing its overall ecological footprint [67]. Addressing these uncertainties requires comprehensive studies across different agricultural contexts to evaluate melanin’s interactions with soil and ecosystems over time. Implementing precautionary measures and monitoring protocols could mitigate potential risks and ensure responsible use in agriculture, balancing innovation with environmental stewardship to support sustainable farming practices.

Stability and degradation: The stability and degradation of melanin under different environmental conditions, such as temperature, pH, and UV exposure, pose significant considerations in its agricultural application. Melanin is known for its robustness and resistance to degradation, yet its stability can vary depending on environmental factors. Exposure to extreme temperatures or fluctuations in pH levels may alter melanin’s structure and functionality, potentially impacting its efficacy as a soil conditioner or biocontrol agent. Moreover, prolonged exposure to UV radiation could accelerate melanin degradation, leading to the formation of breakdown products whose effects on soil health and plant growth remain poorly understood [68].

Understanding the fate of melanin and its degradation products in agricultural ecosystems is crucial for assessing potential risks and benefits. Research efforts should focus on elucidating the mechanisms of melanin degradation under realistic agricultural conditions and evaluating its long-term impacts on soil microbial communities, nutrient cycling, and overall ecosystem resilience. This knowledge gap underscores the need for comprehensive studies that integrate laboratory experiments with field-scale assessments to simulate real-world scenarios. By examining melanin’s stability and degradation dynamics comprehensively, stakeholders can make informed decisions regarding its sustainable use in agriculture, balancing innovation with environmental stewardship.

Unknown effects on crop quality: The potential effects of melanin on the nutritional and commercial quality of crops present a significant knowledge gap in agricultural research. While melanin is primarily considered inert and biocompatible, its application to crops could potentially influence key quality attributes such as taste, texture, and shelf life. These effects may arise from interactions between melanin residues and plant metabolism, affecting the biochemical pathways involved in flavor development, fruit ripening, or post-harvest physiology. Furthermore, melanin’s ability to interact with the moisture and gases present in storage conditions could impact the preservation and overall quality retention of agricultural products [69].

The thorough assessment of melanin’s impact on crop quality requires comprehensive studies that integrate field trials, sensory evaluations, and biochemical analyses. Understanding the mechanisms by which melanin interacts with crop tissues and metabolic processes is essential for predicting and mitigating any adverse effects on taste, texture, or shelf life. Such research efforts are crucial to ensure that melanin-based agricultural practices contribute positively to food quality and safety standards, aligning with consumer preferences and market demands for high-quality produce.

## 5. Prospects of Melanin in Agriculture

Melanin holds promising prospects in agriculture due to its unique properties and potential applications across various agricultural domains. Firstly, melanin exhibits robustness and resistance to degradation, which can enhance its durability and longevity in agricultural settings, potentially improving soil health and plant resilience. It has been explored for its ability to enhance soil structure and water retention, which are critical for sustaining plant growth in diverse environmental conditions [7,66,68].

Moreover, melanin’s natural ability to absorb and dissipate UV radiation makes it a promising candidate for protecting crops from UV damage, thereby reducing stress and improving crop yield and quality. Additionally, melanin has shown potential as a biocontrol agent against plant pathogens, offering sustainable alternatives to chemical pesticides and fungicides. Its ability to interact with microbial communities in the soil may also contribute to ecosystem balance and resilience [7,68,70].

Furthermore, melanin’s biocompatibility and minimal environmental impact underscore its potential for use in organic and sustainable agriculture practices. As research continues to explore its applications, including its potential role in enhancing nutrient uptake or improving post-harvest shelf life, melanin stands poised to contribute to the advancement of agricultural technologies aimed at increasing productivity while minimizing environmental impact [68,70,71].

Overall, while challenges such as production scalability and regulatory considerations remain, melanin represents a versatile and promising avenue for innovation in agriculture, offering solutions that align with modern agricultural sustainability goals [7,63,66,69].

## 6. Conclusions and Outlook

**Figure 2:**
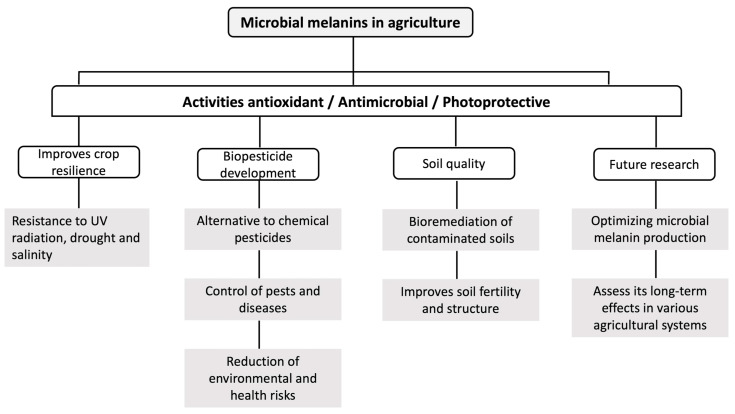
Agricultural applications of microbial melanins.

Microbial melanins are a group of pigments with photoprotective, antimicrobial, and antioxidant activities. These pigments are present in fungi and bacteria, fulfilling different roles. According to their properties, microbial melanins offer a wide range of agricultural applications with the potential to improve crop resilience, soil quality, and integrated pest and disease management practices (Figure 2). Future research should focus on optimizing microbial melanin production and assess its long-term effects in various agricultural systems. Integrating microbial melanin into agricultural biotechnology promises to significantly contribute to more sustainable and resilient agriculture in the face of current and future environmental challenges.

Melanin offers substantial potential for enhancing crop resilience against various abiotic stresses. Its antioxidant and UV-protectant properties can help crops withstand extreme environmental conditions such as high UV radiation, drought, and salinity. The application of microbial melanin could lead to the development of biostimulants that fortify plants against these stresses, ensuring stable yields and improved crop health in challenging climates.

The antimicrobial properties of microbial melanin suggest its use in developing biopesticides. These natural, environmentally friendly alternatives to chemical pesticides could help manage pests and diseases more sustainably. Microbial melanin-based biopesticides can reduce the adverse environmental and health hazards associated with traditional pesticides by decreasing the dependence on synthetic chemicals.

Microbial melanin has the potential to improve soil health significantly. Its ability to chelate heavy metals and other contaminants can aid in the bioremediation of polluted soils, restoring their fertility and functionality. Furthermore, melanin can influence nutrient dynamics in the soil, enhancing the availability of essential nutrients such as iron and phosphorus. Plant nutrition and soil structure can be improved, thus contributing to overall agricultural productivity.

Further research is needed to fully harness microbial melanin’s potential in agriculture. This potential includes optimizing microbial production methods to increase yield and reduce costs and conducting field trials to evaluate the efficacy of melanin-based products under various agricultural conditions. Additionally, studies concerning the long-term environmental impact of melanin applications are crucial to ensure sustainable use.
